# Assessment of routine pre-operative group and save testing in patients undergoing cholecystectomy: a retrospective cohort study

**DOI:** 10.3310/nihropenres.13543.1

**Published:** 2024-04-05

**Authors:** Lawrence O'Leary, William B Sherwood, Michael G Fadel, Musa Barkeji

**Affiliations:** 1 Department of General Surgery, West Middlesex University Hospital, London, TW7 6AF, UK; 2 Department of Pharmacology and Therapeutics, Institute of Systems, Molecular and Integrative Biology, University of Liverpool, Liverpool, L69 3GE, UK; 3 Department of Surgery and Cancer, Imperial College London, London, W12 0NN, UK; 4 Department of General Surgery, Chelsea and Westminster Hospital, London, SW10 9NH, UK

**Keywords:** Blood transfusion, blood typing, cholecystectomy, crossmatching, gallstones, group and save, haemorrhage, type and save

## Abstract

**Background:**

Routine group and save (G&S) testing is frequently performed prior to cholecystectomy, despite growing evidence that a targeted approach is safe and avoids unnecessary investigations. This retrospective cohort study explored frequency of testing in our unit, and rates of and independent pre-operative risk factors for peri-operative blood transfusion.

**Methods:**

Health records of 453 consecutive adults who underwent cholecystectomy in a UK NHS trust were reviewed for blood transfusion up to 30 days post-operatively. We compared the need for transfusion against patient demographics, indication and urgency of surgery, and the number of prior emergency hospital attendances with gallstone complications. Logistic regression determined whether prior attendances with complications of gallstones independently predicted the need for transfusion.

**Results:**

Peri-operative blood transfusions within 30 days of operation occurred in 1.1% of cases, with no requirement for uncrossmatched blood. Patients who received a blood transfusion tended to have higher American Society of Anesthesiologists (ASA) grades ( *p*<0.001), were more likely to have an underlying primary haematological malignancy (20.0% vs. 0.2%; *p*<0.001) and prior emergency hospital attendances with gallstone complications (median 4 vs. 1; *p*<0.001). Logistic regression showed each prior emergency attendance was associated with 4.6-fold odds of transfusion ( *p*=0.019). Receiver operating characteristic curve analysis showed an area under the curve of 0.92. Three or more attendances predicted need for transfusion with 60.0% sensitivity and 98.0% specificity. Seventy-four percent had at least one G&S sample taken pre-operatively, costing the trust approximately £3,800 per year in materials.

**Conclusions:**

Pre-operative G&S testing prior to cholecystectomy may not be routinely required. Increased frequency of prior emergency hospital attendances with gallstone complications and co-morbidities associated with coagulopathies were pre-operative risk factors for post-operative blood transfusion. More selective testing could provide large financial savings for health institutions without compromising patient safety.

## Plain english summary

Many patients diagnosed with gallstones undergo surgery to remove their gallbladder. Blood tests are routinely performed in clinic before the operation, which may include testing a patient’s blood type in case of bleeding during or after the operation. This test is required before a patient receives a blood transfusion.

Testing the blood type of patients prior to gallbladder surgery may not be routinely required, as excessive bleeding from this operation is rare. Avoiding unnecessary blood tests protects patients from unwarranted needles, prevents delays to surgery and saves hospitals money. We therefore investigated how frequently patients undergoing gallbladder surgery in our unit have blood type testing. We also assessed how often patients require a blood transfusion and whether testing for blood type could have been safely performed once the need for transfusion was identified, instead of before the operation.

Looking back at 453 patients’ notes, we found that only five needed a blood transfusion within 30 days of their operation. None of these blood transfusions were needed on an emergency basis, and therefore there would have been enough time to test these patients for their blood type without having to test them before the operation. Despite this, nearly three-quarters of our patients had blood type testing before the operation. Each of these tests costs an estimated £11 per sample in our unit, and therefore £3,800 per year could have been saved were these tests not performed.

The five patients who required a transfusion were found to have attended hospital more times before their operation with problems related to gallstones. Therefore, our study provides evidence that performing gallbladder operations on a more urgent basis following an admission to hospital with gallstone complications could reduce a patients’ risk of bleeding.

## Introduction

Pre-operative evaluation is an opportunity to assess and mitigate a patient’s risk of undergoing anaesthesia and surgery. Blood tests are a common component of this assessment and may include group and save (G&S) testing, which identifies a patient’s red blood cell type and screens for irregular antibodies. It has been suggested that G&S testing is overperformed prior to laparoscopic cholecystectomy and a targeted approach should be adopted ^1– 14^. Yet the United Kingdom (UK) lacks national guidance recommending which patient or procedural factors warrant pre-operative G&S testing.

Guidelines published in 2016 by the UK National Institute for Health and Care Excellence (NICE) on pre-operative testing prior to elective surgery stratified patients by their co-morbidities and the complexity of the proposed surgery ^15^. The aim was to standardise and avoid unnecessary investigations in low- and intermediate-risk operations, however the committee excluded from the scope of the guidelines specific advice relating to G&S testing. Guidelines provided by the French Society of Anaesthesiology and Intensive Care advise if the risk of bleeding or blood transfusion is perceived to be “nil to low”, G&S testing is unnecessary, whereas if it is “intermediate to high”, these tests should be performed ^16^. However, they do not give specific advice as to which patient and operative factors should be considered as part of this risk assessment.

Choosing Wisely, an international campaign to reduce unnecessary investigations, published recommendations from the Canadian Society of Transfusion Medicine that included specific advice to avoid routine G&S testing in patients undergoing cholecystectomy, as transfusion rates among these patients are low ^17^. This advice is infrequently heeded: we recently published a systematic review of the available literature relating to G&S testing in patients who underwent cholecystectomy ^1^. Over 460,000 patients were included, 37.8% of whom underwent pre-operative G&S testing. Peri-operative transfusions occurred in only 2.1% of patients, and the authors of all the studies included in the review recommended a more targeted approach to pre-operative G&S testing ^1– 14^.

The decision to perform G&S testing relies on the clinical judgement of staff running pre-operative assessment clinics. For fully crossmatched blood to be issued in our unit, patients must have one G&S sample taken within the preceding five days, plus a second sample that can be of any age on the patient’s electronic health records. Without clear guidance, inexperienced staff may err on the side of over-investigation ^18^. Many patients will have two samples taken pre-operatively due to the erroneous perception that this would decrease the time for crossmatched blood to be issued in the unlikely event of rapid intra-operative haemorrhage. As Ghirardo and his colleagues pointed out, such an event would demand rapid transfusion with uncrossmatched blood products, during which time G&S testing can be performed and crossmatched blood obtained within twenty minutes ^6^. Even if G&S samples were taken pre-operatively, the crossmatching of blood products would take the same amount of time in this scenario.

Purported pre-operative risk factors for peri-operative blood transfusion in the available literature include anaemia, regular oral anticoagulant use, cardiovascular co-morbidities, type 2 diabetes mellitus, primary haematological malignancies and increased American Society of Anesthesiologists (ASA) grade ^5, 6, 9, 11, 14^. None of these authors have assessed whether these associations are independent predictors of blood transfusion.

NICE and international guidelines support urgent cholecystectomy over a planned, delayed operation in those presenting with acute biliary pathology (especially cholecystitis and gallstone pancreatitis), as it reduces length of hospital stay, medical costs, number of workdays lost, and prevents recurrent admissions whilst on a waiting list ^19– 22^. A large population-based study in Canada showed patients with acute cholecystitis who were discharged without early cholecystectomy had a 14% chance of acute hospital attendance with gallbladder pathology within 6 weeks or 29% within a year ^23^. Despite this, there is great variability in inter-hospital practice regarding the proportion of cholecystectomies performed on an emergency basis ^24^. Repeated episodes of acute biliary pathology could increase operative difficulty, and hence increase the risk of bleeding and need for blood transfusion.

This study audited our unit’s peri-operative blood transfusion and G&S testing rates in patients undergoing cholecystectomy. It aimed to identify independent pre-operative risk factors that may predict peri-operative blood transfusion in these patients. We also hypothesised that the odds of transfusion could be predicted by the number of prior emergency attendances to hospital with acute biliary pathology secondary to gallstones. To our knowledge, we are the first to explore this association. Armed with this, and with a greater understanding of local transfusion rates and the financial implications of routine G&S testing, we aim to improve selectivity of pre-operative G&S testing prior to this very common procedure.

## Methods

### Patient and public involvement

This project was born out of several informal discussions with patients pre-operatively. Some were found not to have two valid group and save samples on the mornings of their operations, and some senior members of the theatre team insisted the case could not go ahead until this had been rectified. We identified a lack of local or national guidance to advise on the matter, which led to variability in the specific blood tests taken at patients’ pre-operative clinic appointments. Many patients expressed frustration at the need to undergo additional venipuncture and at the delay to their operation until the blood tests had been taken. Occasionally, this led to some patients undergoing an intended day-case procedure staying overnight due to late starts to their procedures.

There was universal support from these patients for a project that would help to create a consensus on the pre-operative blood tests performed prior to routine elective surgery with low risk of blood transfusion. We are very grateful for sharing their comments and opinions with the authors of this study.

### Study methodology

This cross-sectional study analysed 453 consecutive adult patients who underwent laparoscopic or open cholecystectomy between August 2019 and January 2021 in one NHS trust in London, UK. The trust comprises two large teaching hospitals, each with an accident and emergency department and a general surgical department.

Patients were identified by their International Classification of Diseases, 10 ^th^ revision, codes. Data were extracted from patients’ electronic hospital records and anonymised. The database was registered locally as a clinical audit. Patients aged under 16 years were not included in the study. Patient notes were analysed for receipt of transfusion of packed red blood cells either intra-operatively or up to 30 days post-operatively.

For a patient to have two valid G&S samples pre-operatively, one sample would need to be taken within the preceding five days of their operation, and two samples if the patient did not have a prior G&S sample on their hospital electronic records. To provide a cost-analysis for the processing of pre-operative G&S samples, we present the number of G&S samples taken within five days of the operation. We have not included the cost of historic samples on patients’ electronic records.

Indication for surgery was defined as the diagnosis made at the patient’s last emergency hospital attendance, or from that stated on the patient’s latest general surgical clinic letter if that patient had no prior emergency attendances. An emergency operation was defined as cholecystectomy undertaken during the same admission as an emergency attendance to the hospital; otherwise, the procedure was considered having been performed electively.

Length of hospital stay referred to the number of post-operative days during the admission in which the operation took place. It did not include any prior admissions nor post-operative readmissions.

A patient was deemed to have cardiovascular co-morbidities if at least one of the following were recorded in the patient’s electronic health records: hypertension, ischaemic heart disease, peripheral vascular disease, atrial fibrillation, or cerebrovascular accident.

Estimated intra-operative blood loss was recorded by the operating surgeon in the operation note. If the surgeon had written “minimal” or similar, a value of 0 ml was assigned for the purpose of analysis.

Pre-operative haemoglobin was the most recent pre-operative blood test performed; post-operative haemoglobin was the lowest value within 30 days of the operation. All patients had their pre-operative haemoglobin level checked. One hundred and ninety-five (43.0%) patients had their post-operative haemoglobin level checked. Analyses on pre- to post-operative haemoglobin change therefore excluded those patients who did not have a post-operative haemoglobin check. All other data recorded in this study were complete.

An emergency attendance with gallstones complications was defined according to diagnosis on discharge as one of the following: biliary colic, acute cholecystitis, pancreatitis, obstructive jaundice, gallbladder polyps or empyema. Prior attendances with any other pathology were disregarded.

### Statistical analysis

Data analyses were performed using IBM SPSS Statistics v.28 (IBM, Armonk, New York, USA). A free software alternative is GNU PSPP v.2.0.0 (Free Software Foundation, Boston, Massachusetts, USA). Statistical analyses of categorical data were performed using Pearson’s chi-squared test. We present the chi-squared value with the number of degrees of freedom in brackets. Differences between numerical data were explored using the Mann-Whitney *U* test. A *p*-value less than 0.05 was considered statistically significant.

Binomial logistic regression determined whether the number of emergency attendances to the same hospital trust in the preceding three years with complications of gallstones predicted risk of requiring a red blood cell transfusion within 30 days of cholecystectomy independent of age, gender, urgency of operation, indication for surgery, ASA grade, presence of cardiovascular co-morbidities, type 2 diabetes mellitus, primary haematological malignancy, regular use of oral anticoagulants or antiplatelets, and pre-operative haemoglobin level. The Wald chi-squared test was used to determine statistical significance for each of the independent variables and is presented along with the number of degrees of freedom in brackets.

To assess further the power that frequency of emergency hospital attendances predicts post-operative blood transfusion, a receiver operating characteristic (ROC) curve was drawn. The area under the curve (AUC) can be used to visualise the sensitivity and specificity of a model predicting a certain outcome: the closer the AUC is to 1, the more accurately the model correctly classifies outcomes. Here, we present the AUC with 95% confidence intervals.

### Statement of ethics

This study and its database were registered as a clinical audit and approved locally by the West Middlesex University Hospital audit department (audit registration number PCD935). All data were anonymised and collated as part of a clinical audit and therefore patient consent was not required.

## Results

Our data have been made publicly available on Open Science Framework at https://osf.io/5uva4/ ^25^.

Five of the 453 patients (1.1%) received a blood transfusion within 30 days of their operation. Table 1 summarises demographic characteristics of the patients, stratified by whether they received a blood transfusion. Those who received a transfusion tended to be more co-morbid, as measured by ASA grade (χ ^2^ (3) = 90.3, *p* < 0.001). One patient in each group had an underlying primary haematological malignancy predisposing to bleeding, which was found to be a statistically significant risk factor for receiving a blood transfusion (χ ^2^ (1) = 44.0, *p* < 0.001): the patient who received a transfusion had myelodysplasia characterized by chronic anaemia and thrombocytopaenia. Similar rates of cardiovascular co-morbidities (χ ^2^ (1) = 0.2, *p* = 0.630) and type 2 diabetes mellitus (χ ^2^ (1) = 0.4, *p* = 0.548) were seen between the two groups.

**Table 1.** Patient demographics stratified by whether they received a blood transfusion. Data are presented as number of patients, followed by the percentage of patients within that transfusion status group in brackets, unless specified otherwise. ASA, American Society of Anesthesiologists; G&S, Group and Save; Hb, Haemoglobin; IQR, InterQuartile Range; NA, Not Applicable; *Mann-Whitney *U* test. †Pearson’s chi-square test.

Demographic	Blood transfusion received	Blood transfusion not received	*p*-value
**Patients, *n* (% of total)**	5 (1.1)	448 (98.9)	NA
**Median age, years (IQR)**	59 (34 – 67)	50 (39 – 61)	0.703 *
**Gender** ** Female** ** Male**	4 (80.0) 1 (20.0)	308 (68.8) 140 (31.3)	0.589 ^†^
**Median body mass index, kg/m ^2^ (IQR)**	25 (24 – 31)	28 (25 – 32)	0.425 *
**ASA grade** ** I** ** II** ** III** ** IV** ** V**	1 (20.0) 3 (60.0) 0 (0.0) 1 (20.0) 0 (0.0)	128 (28.6) 272 (60.7) 48 (10.7) 0 (0.0) 0 (0.0)	< 0.001 ^†^
**Co-morbidities** ** Cardiovascular** ** Type 2 diabetes mellitus** ** Primary haematological malignancy** ** Regularly on oral anticoagulant or antiplatelet**	1 (20.0) 1 (20.0) 1 (20.0) 0 (0.0)	134 (29.9) 51 (11.4) 1 (0.2) 33 (7.4)	0.630 ^†^ 0.548 ^†^ < 0.001 ^†^ 0.529 ^†^
**Indication** ** Biliary colic** ** Acute cholecystitis** ** Pancreatitis** ** Obstructive jaundice** ** Gallbladder polyps** ** Empyema**	1 (20.0) 3 (60.0) 0 (0.0) 1 (20.0) 0 (0.0) 0 (0.0)	217 (48.4) 132 (29.5) 45 (10.0) 27 (6.0) 12 (2.7) 15 (3.3)	0.448 ^†^
**Operative urgency** ** Emergency** ** Elective**	1 (20.0) 4 (80.0)	80 (17.9) 368 (82.1)	0.901 ^†^
**Operation type** ** Completed laparoscopically** ** Laparoscopic converted to open Primary open**	5 (100.0) 0 (0.0) 0 (0.0)	444 (99.1) 3 (0.7) 1 (0.2)	0.978 ^†^
**Median estimated intra-operative blood loss, ml (IQR)**	0 (0 – 50)	0 (0 – 0)	0.056 *
**Median pre-operative Hb, g/l (IQR)**	132 (121 – 145)	134 (126 – 143)	0.651 *
**Median change between pre- and post-operative Hb, g/l (IQR)**	-36 (-68 – -35)	-8 (-14 – -2)	0.001 *
**Number of pre-operative G&S samples** ** 0** ** 1** ** 2**	0 (0.0) 1 (20.0) 4 (80.0)	116 (25.6) 178 (39.5) 154 (34.9)	0.094 ^†^
**Median length of hospital stay, days (IQR)**	8 (5 – 12)	1 (0 – 2)	0.002 *
**Median number of prior emergency hospital attendances** ** with complications of gallstones, *n* (IQR)**	4 (2 – 4)	1 (0 – 1)	< 0.001 *

As would be expected, those patients requiring a blood transfusion had greater drops in post-operative haemoglobin (-36 g/l vs. -8 g/l, *U* = 77, *p* = 0.001), though the two groups started with similar pre-operative haemoglobin levels (132 g/l vs. 134 g/l, *U* = 957.5, *p* = 0.651). Blood transfusions were associated with longer inpatient stays (median 8 days vs. 1 day, *U* = 268, *p* = 0.002).

The patient characteristics and indications for transfusion for each of the five cases who received a blood transfusion are detailed in the Supplementary Table. None required emergency O negative blood intra-operatively or post-operatively. At the time of identification of the need for a blood transfusion, each patient was haemodynamically stable; as such, sufficient time would have been present for two G&S samples to be taken and for blood to be fully crossmatched prior to transfusion.

In 116 (25.6%) cases, no G&S samples were taken in the preceding five days of the operation. One hundred and seventy-nine (39.5%) patients had one G&S sample taken and 158 (34.9%) had two. 301 of the 404 patients with ASA grade I or II had at least one G&S sample taken. The cost of analysing a G&S sample in our trust is £11, excluding laboratory staffing costs. Within our cohort, analysis of pre-operative G&S samples therefore cost in the region of £3,800 per year in materials.

Patients who required a peri-operative blood transfusion had more prior emergency hospital attendances in the preceding three years with gallstone complications (median 4 attendances vs. 1 attendance, *U* = 186, *p* < 0.001). A logistic regression was performed to determine whether this association held independently of the effects of age, gender, urgency of operation, indication, ASA grade, presence of cardiovascular co-morbidities, type 2 diabetes mellitus, primary haematological malignancy, regular oral anticoagulant or antiplatelet use, or pre-operative haemoglobin level. The regression model was statistically significant (χ ^2^ (17) = 28.3, *p* = 0.042), explained 53.3% (Nagelkerke *R* ^2^) of the variance in blood transfusion requirement and correctly classified 98.9% of cases. Following adjustment for the other co-variants, we found that with each additional emergency hospital attendance, the odds of requiring a blood transfusion increased by a factor of 4.6 (95% confidence interval 1.3 to 16.3, Wald χ ^2^ (1) = 5.5, *p* = 0.019). No other co-variants added significantly to the model.

**Figure 1.**
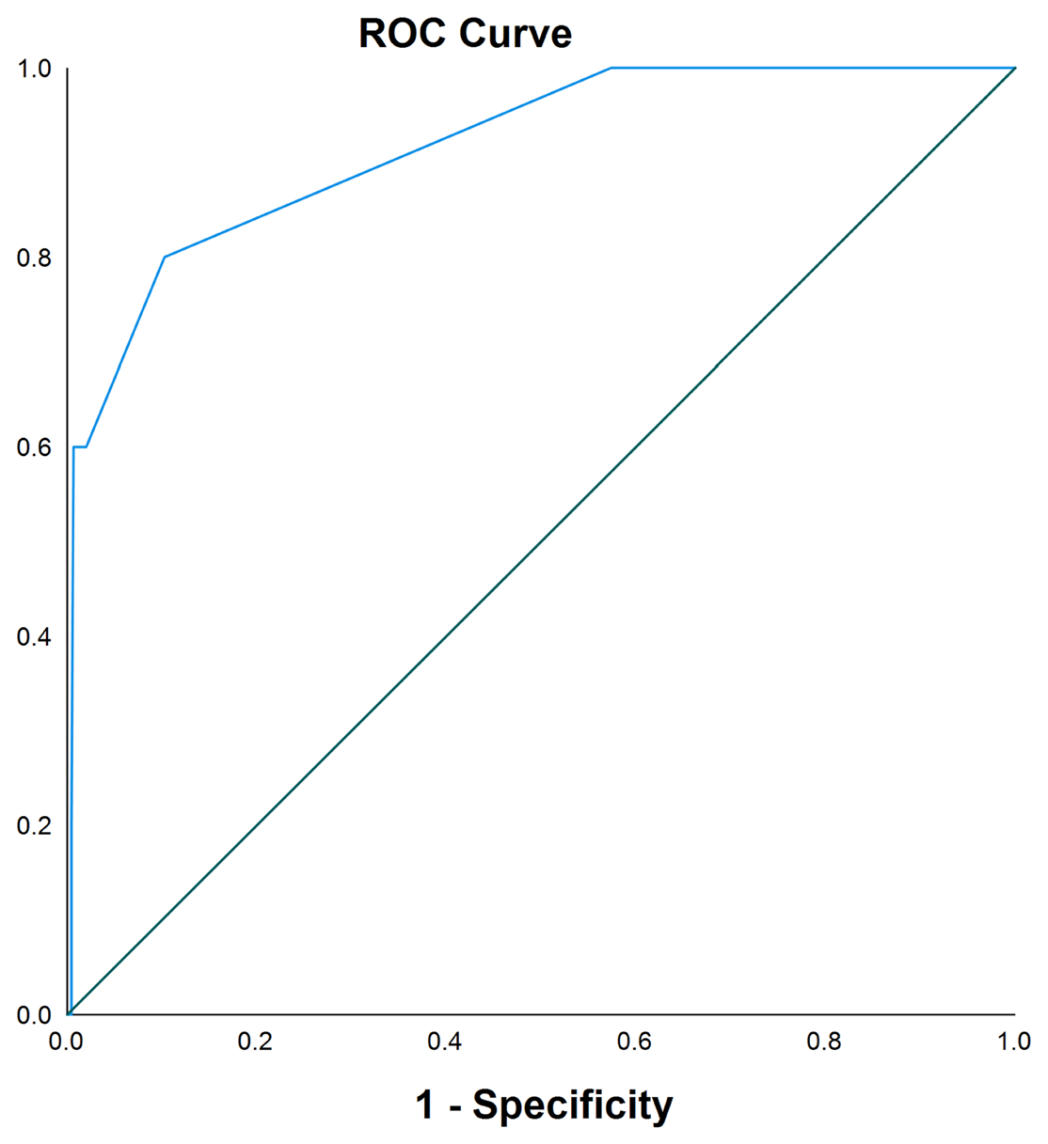
Receiver Operating Characteristic (ROC) curve analysis of number of prior emergency hospital attendances and its impact on the requirement for post-operative blood transfusion. Area under the curve 0.92 (95% confidence interval 0.79 – 1.00).

ROC curve analysis showed an AUC of 0.92 (95% confidence interval 0.79 – 1.00) for number of prior emergency hospital attendances as a predictor of the need for post-operative blood transfusion ( Figure 1). Three or more prior attendances predicted those patients who would require a post-operative blood transfusion with 60.0% sensitivity and 98.0% specificity.

## Discussion

To our knowledge, we are the first to demonstrate that the number of pre-operative emergency attendances with acute gallstone complications can be a strong independent predictor of the need for a post-operative blood transfusion following cholecystectomy. Within our cohort, three or more emergency hospital attendances with biliary pathology was highly predictive of this outcome. Indeed, this was the only pre-operative factor that we explored demonstrating a statistically significant independent association following logistic regression. It is likely that frequent flare-ups of biliary inflammation creates more local fibrosis and adhesions, setting up a technically more challenging procedure, as was found in Lo and colleagues’ randomised trial of early versus delayed cholecystectomies ^26^. Fibrosis and oedema in chronic cholecystitis destroy clear operative planes between the gallbladder and the liver bed, which may increase the risk of bleeding from the raw liver surface ^27^. Increased vascular fragility and bleeding may result from the vascular congestion and angiogenesis associated with chronic inflammatory processes ^27, 28^. Four of the 5 patients who required a blood transfusion in our cohort developed post-operative subhepatic haematomas. It may be that these cases were technically challenging, and therefore achieving adequate haemostasis was more difficult; or, even if haemostasis were achieved initially, a high risk of rebleeding followed abdominal closure.

In 1995, Nassar and colleagues published a grading system classifying laparoscopic cholecystectomies according to intra-operative technical difficulty, taking into consideration the density of adhesions and features of the gallbladder and cystic pedicle that make for a more complex dissection ^29^. Their team have shown that patients who undergo emergency laparoscopic cholecystectomy are more likely to have a higher grade of difficulty if they have had multiple hospital admissions with acute biliary disease compared to those for operated on in their first admission, and that a higher Nassar scale grade is associated with a higher risk of bleeding ^30, 31^.

In one case (Case 3, Supplementary Table), the need for post-operative blood transfusion was attributed to underlying primary haematological malignancy. Granted, the number of patients with this co-morbidity in our cohort is small, but its association with receiving a blood transfusion reached statistical significance, though our regression model did not show that this was an independent predictor of transfusion. Others have also found primary haematological cancers to be a risk factor for peri-operative transfusion: in an observational study of 4,462 patients in Scotland, this co-morbidity was present in 6 of the 48 (12.5%) cases who required a blood transfusion ^11^. A single-centre retrospective study of 1,167 patients undergoing cholecystectomy in the USA also found that one of their five patients who required a peri-operative blood transfusion had a diagnosis of chronic leukaemia with thrombocytopaenia ^6^. We also found an association between higher ASA grade and need for post-operative transfusion in cholecystectomy, confirming findings by other groups, though this association also did not hold through our regression model ^9, 11^.

Other cohort studies have found associations between pre-operative anaemia, obstructive jaundice as an indication for surgery, regular oral anticoagulant use, and underlying type 2 diabetes mellitus ^5, 6, 9, 11, 14^. Our study did not show these to be predictors of post-operative blood transfusion. This may be owing to the rarity of patients requiring blood transfusion in our cohort, and a more highly powered study may be required to confirm or refute these associations.

Our study also did not correlate presence of cardiovascular co-morbidities with the need for blood transfusion. Wan and colleagues noted that many post-cholecystectomy patients developed a drop in haemoglobin and haematocrit, even when intra-operative overt blood loss was negligible ^32^. Seeking factors that could be attributable to this “hidden blood loss”, they found that patients with a history of controlled hypertension had over 130 millilitres’ greater estimated blood loss than those with no history of hypertension. The authors suggested that people with chronic hypertension may have more fragile vessels that predispose to post-operative bleeding. Relating these findings to our cohort, perhaps the increased blood loss, overt or hidden, associated with cardiovascular disease is insufficient to warrant a transfusion.

At 1.1%, peri-operative blood transfusion following cholecystectomy in our cohort is rare, and comparable to that in the available literature ^1– 14^. In all of our cases, following identification of the need for peri-operative blood transfusion, there is usually enough time for two G&S samples to be taken, typed and fully crossmatched blood procured, which corroborates the findings of other cohort studies ^5, 6, 12^. In the rare cases of intra-operative major vascular injury, it would be detrimental to the patient to await crossmatched blood, and therefore even with two valid G&S samples available, rapid transfusion of O negative blood would be required ^1, 6, 12, 14^. G&S testing pre-operatively is therefore not required in most cases ^1– 14^.

There are important limitations that must be considered when interpreting the study findings. This study was retrospective in nature with an adequate sample size over a 19-month period. The main number of patients included is comparable to many other cohort studies investigating rates of peri-operative blood transfusion, fortunately the need for blood products following cholecystectomy is rare ^2, 8, 9, 13^. Therefore, where associations between risk factors and blood transfusion are statistically insignificant, it can be difficult to refute a correlation that may not be seen due to underpowering of our study. Future prospective national studies, with a larger sample size, is required to make a further assessment of whether G&S routine testing is required and its associated safety.

## Conclusions

Our study suggests that routine G&S testing prior to cholecystectomy, despite this being common practice in many units, may not be essential. A more selective approach to pre-operative G&S testing would be safe and avoid wasting resources. It can also be inferred that performing cholecystectomy on an urgent basis following emergency admission with acute biliary pathology should be considered where possible to avoid recurrent episodes, as the frequency of prior emergency hospital attendances with gallstone complications increases the risk of receiving post-operative blood transfusion. Further research is required to determine a specific patient selection criteria for G&S testing in other surgical specialties.

## Data Availability

Open Science Framework: Underlying data for ‘Assessment of routine pre-operative group and save testing in patients undergoing cholecystectomy: a retrospective cohort study’, https://doi.org/10.17605/OSF.IO/84HTQ ^25^ Data are available under the terms of the Creative Commons Zero “No rights reserved” data waiver (CC0 1.0 Public domain dedication).
