# Assessment of routine pre-operative group and save testing in patients undergoing cholecystectomy: a retrospective cohort study

**DOI:** 10.3310/nihropenres.13543.2

**Published:** 2024-10-23

**Authors:** Lawrence O'Leary, William B Sherwood, Michael G Fadel, Musa Barkeji

**Affiliations:** 1 Department of General Surgery, West Middlesex University Hospital, London, TW7 6AF, UK; 2 Department of Pharmacology and Therapeutics, Institute of Systems, Molecular and Integrative Biology, University of Liverpool, Liverpool, L69 3GE, UK; 3 Department of Surgery and Cancer, Imperial College London, London, W12 0NN, UK; 4 Department of General Surgery, Chelsea and Westminster Hospital, London, SW10 9NH, UK

**Keywords:** Blood transfusion, blood typing, cholecystectomy, crossmatching, gallstones, group and save, haemorrhage, type and save

## Abstract

**Background:**

Routine group and save (G&S) testing is frequently performed prior to cholecystectomy, despite growing evidence that a targeted approach is safe and avoids unnecessary investigations. This retrospective cohort study explored frequency of testing in our unit, rates of peri-operative blood transfusion and pre-operative risk factors for requiring transfusion.

**Methods:**

Health records of 453 consecutive adults who underwent cholecystectomy in a UK NHS trust were reviewed for blood transfusion up to 30 days post-operatively. We compared the need for transfusion against patient demographics, indication and urgency of surgery, and the number of prior emergency hospital attendances with gallstone complications. Logistic regression determined whether prior attendances with complications of gallstones independently predicted the need for transfusion.

**Results:**

Peri-operative blood transfusions within 30 days of operation occurred in 1.1% of cases, with no requirement for uncrossmatched blood. Patients who received a blood transfusion tended to have higher American Society of Anesthesiologists (ASA) grades ( *p* = 0.017), were more likely to have an underlying primary haematological malignancy (20.0% vs. 0.2%; *p* = 0.022) and prior emergency hospital attendances with gallstone complications (median 4 vs. 1; *p* < 0.001). Logistic regression showed each prior emergency attendance was associated with 4.6-fold odds of transfusion ( *p* = 0.019). Receiver operating characteristic curve analysis showed an area under the curve of 0.92. Three or more attendances predicted need for transfusion with 60.0% sensitivity and 98.0% specificity. 74% of patients had at least one G&S sample taken pre-operatively, costing the trust approximately £3,800 per year in materials.

**Conclusions:**

The findings of this study suggest that pre-operative G&S testing prior to cholecystectomy is not routinely required. Increased frequency of prior emergency hospital attendances with gallstone complications and co-morbidities associated with coagulopathies were pre-operative risk factors for post-operative blood transfusion. More selective testing could provide large financial savings for health institutions without compromising patient safety.

## Plain english summary

Many patients diagnosed with gallstones undergo surgery to remove their gallbladder. Blood tests are routinely performed in clinic before the operation, which may include testing a patient’s blood type in case of bleeding during or after the operation. This test is required before a patient receives a blood transfusion.

Testing the blood type of patients prior to gallbladder surgery may not be routinely required, as excessive bleeding from this operation is rare. Avoiding unnecessary blood tests protects patients from unwarranted needles, prevents delays to surgery and saves hospitals money. We therefore investigated how frequently patients undergoing gallbladder surgery in our unit have blood type testing. We also assessed how often patients require a blood transfusion and whether testing for blood type could have been safely performed once the need for transfusion was identified, instead of before the operation.

Looking back at 453 patients’ notes, we found that only five needed a blood transfusion within 30 days of their operation. None of these blood transfusions were needed on an emergency basis, and therefore there would have been enough time to test these patients for their blood type without having to test them before the operation. Despite this, nearly three-quarters of our patients had blood type testing before the operation. Each of these tests costs an estimated £11 per sample in our unit, and therefore £3,800 per year could have been saved were these tests not performed.

The five patients who required a transfusion were found to have attended hospital more times before their operation with problems related to gallstones. Therefore, our study provides evidence that performing gallbladder operations on a more urgent basis following an admission to hospital with gallstone complications could reduce a patients’ risk of bleeding.

## Introduction

Pre-operative evaluation is an opportunity to assess and mitigate a patient’s risk of undergoing anaesthesia and surgery. Blood tests are a common component of this assessment and may include group and save (G&S) testing, which identifies a patient’s red blood cell type and screens for irregular antibodies. It has been suggested that G&S testing is overperformed prior to laparoscopic cholecystectomy and a targeted approach should be adopted ^1– 14^. Yet the United Kingdom (UK) lacks national guidance recommending which patient or procedural factors warrant pre-operative G&S testing.

Guidelines published in 2016 by the UK National Institute for Health and Care Excellence (NICE) on pre-operative testing prior to elective surgery stratified patients by their co-morbidities and the complexity of the proposed surgery ^15^. The aim was to standardise and avoid unnecessary investigations in low- and intermediate-risk operations, however the committee excluded from the scope of the guidelines specific advice relating to G&S testing. Guidelines provided by the French Society of Anaesthesiology and Intensive Care advise if the risk of bleeding or blood transfusion is perceived to be “nil to low”, G&S testing is unnecessary, whereas if it is “intermediate to high”, these tests should be performed ^16^. However, they do not give specific advice as to which patient and operative factors should be considered as part of this risk assessment.

Choosing Wisely, an international campaign to reduce unnecessary investigations, published recommendations from the Canadian Society of Transfusion Medicine that included specific advice to avoid routine G&S testing in patients undergoing cholecystectomy, as transfusion rates among these patients are low ^17^. This advice is infrequently heeded: we recently published a systematic review of the available literature relating to G&S testing in patients who underwent cholecystectomy ^1^. Over 460,000 patients were included, 37.8% of whom underwent pre-operative G&S testing. Peri-operative transfusions occurred in only 2.1% of patients, and the authors of all the studies included in the review recommended a more targeted approach to pre-operative G&S testing ^1– 14^.

The decision to perform G&S testing relies on the clinical judgement of staff running pre-operative assessment clinics. For fully crossmatched blood to be issued in our unit, patients must have one G&S sample taken within the preceding five days, plus a second sample that can be of any age on the patient’s electronic health records. Without clear guidance, inexperienced staff may err on the side of over-investigation ^18^. Many patients will have two samples taken pre-operatively due to the erroneous perception that this would decrease the time for crossmatched blood to be issued in the unlikely event of rapid intra-operative haemorrhage. As Ghirardo and his colleagues pointed out, such an event would demand rapid transfusion with uncrossmatched blood products, during which time G&S testing can be performed and crossmatched blood obtained within twenty minutes ^6^. Even if G&S samples were taken pre-operatively, the crossmatching of blood products would take the same amount of time in this scenario.

Purported pre-operative risk factors for peri-operative blood transfusion in the available literature include anaemia, regular oral anticoagulant use, cardiovascular co-morbidities, type 2 diabetes mellitus, primary haematological malignancies and increased American Society of Anesthesiologists (ASA) grade ^5, 6, 9, 11, 14^. None of these authors have assessed whether these associations are independent predictors of blood transfusion.

NICE and international guidelines support urgent cholecystectomy over a planned, delayed operation in those presenting with acute biliary pathology (especially cholecystitis and gallstone pancreatitis), as it reduces length of hospital stay, medical costs, number of workdays lost, and prevents recurrent admissions whilst on a waiting list ^19– 22^. A large population-based study in Canada showed patients with acute cholecystitis who were discharged without early cholecystectomy had a 14% chance of acute hospital attendance with gallbladder pathology within 6 weeks or 29% within a year ^23^. Despite this, there is great variability in inter-hospital practice regarding the proportion of cholecystectomies performed on an emergency basis ^24^. Repeated episodes of acute biliary pathology could increase operative difficulty, and hence increase the risk of bleeding and need for blood transfusion.

This study audited our unit’s peri-operative blood transfusion and G&S testing rates in patients undergoing cholecystectomy. It aimed to identify independent pre-operative risk factors that may predict peri-operative blood transfusion in these patients. We also hypothesised that the odds of transfusion could be predicted by the number of prior emergency attendances to hospital with acute biliary pathology secondary to gallstones. Armed with this, and with a greater understanding of local transfusion rates and the financial implications of routine G&S testing, we aim to improve selectivity of pre-operative G&S testing prior to this very common procedure.

## Methods

### Patient and public involvement

This project was born out of several informal discussions with patients pre-operatively. Some were found not to have two valid group and save samples on the mornings of their operations, and some senior members of the theatre team insisted the case could not go ahead until this had been rectified. We identified a lack of local or national guidance to advise on the matter, which led to variability in the specific blood tests taken at patients’ pre-operative clinic appointments. Many patients expressed frustration at the need to undergo additional venipuncture and at the delay to their operation until the blood tests had been taken. Occasionally these delays led to some patients staying overnight in hospital, despite their operations having been intended to be day-case procedures.

There was universal support from these patients for a project that would help to create a consensus on the pre-operative blood tests performed prior to routine elective surgery with low risk of blood transfusion. We are very grateful for sharing their comments and opinions with the authors of this study.

### Study methodology

This retrospective cohort study analysed the electronic health records of 453 consecutive adult patients who underwent laparoscopic or open cholecystectomy between August 2019 and January 2021 in one NHS trust in London, UK. Both total and subtotal cholecystectomies were included. The trust comprises two large teaching hospitals, each with an accident and emergency department and a general surgical department.

A database was created by IT professionals in the local audit department, who identified patients by the following Office of Population Censuses and Surveys version 4 (OPCS-4) codes: J18.1 (Total cholecystectomy and excision of surrounding tissue), J18.3 (Total cholecystectomy NEC), J18.4 (Partial cholecystectomy and exploration of common bile duct), J18.5 (Partial cholecystectomy NEC), J18.8 (Other specified excision of gallbladder) and J18.9 (Unspecified excision of gallbladder). Patient notes – which are electronically linked to the transfusion laboratory information system– were analysed and cross-referenced with fluid balance charts for receipt of transfusion of packed red blood cells either intra-operatively or up to 30 days post-operatively.

For a patient to have two valid G&S samples pre-operatively, one sample would need to be taken within the preceding five days of their operation, and two samples if the patient did not have a prior G&S sample on their hospital electronic records. To provide a cost-analysis for the processing of pre-operative G&S samples, we present the number of G&S samples taken within five days of the operation. We have not included the cost of historic samples on patients’ electronic records.

Indication for surgery was defined as the diagnosis made at the patient’s last emergency hospital attendance, or from that stated on the patient’s latest general surgical clinic letter if that patient had no prior emergency attendances. Any patient who presented with obstructive jaundice was investigated with magnetic resonance cholangiopancreatography and, if required, underwent clearance of obstructing choledocholithiasis with endoscopic retrograde cholangiopancreatography prior to cholecystectomy. An emergency operation was defined as cholecystectomy undertaken during the same admission as an emergency attendance to the hospital; otherwise, the procedure was considered having been performed electively.

A patient was deemed to have cardiovascular co-morbidities if at least one of the following were recorded in the patient’s electronic health records: hypertension, ischaemic heart disease, peripheral vascular disease, atrial fibrillation, or cerebrovascular accident. Pre-operative haemoglobin was the most recent pre-operative blood test performed. For those who had their full blood count checked multiple times during the post-operative recovery, we have defined “post-operative haemoglobin” as the lowest value within 30 days of the operation. Estimated intra-operative blood loss was recorded by the operating surgeon in the operation note. If the surgeon had written “minimal” or similar, a value of 0 millilitres was assigned for the purpose of analysis.

### Statistical analysis

Data analyses were performed using IBM SPSS Statistics v.28 (IBM, Armonk, New York, USA). A free software alternative is GNU PSPP v.2.0.0 (Free Software Foundation, Boston, Massachusetts, USA). Statistical analyses of categorical data were performed using Fisher’s exact test. Differences between numerical data were explored using the Mann-Whitney *U* test. A *p*-value less than 0.05 was considered statistically significant.

Binomial logistic regression determined whether the number of emergency attendances to the same hospital trust in the preceding three years with complications of gallstones predicted risk of requiring a red blood cell transfusion within 30 days of cholecystectomy independent of age, gender, urgency of operation, indication for surgery, ASA grade, presence of cardiovascular co-morbidities, type 2 diabetes mellitus, primary haematological malignancy, regular use of oral anticoagulants or antiplatelets, and pre-operative haemoglobin level. The Wald χ ^2^ test was used to determine statistical significance for each of the independent variables.

To assess further the power that frequency of emergency hospital attendances predicts post-operative blood transfusion, a receiver operating characteristic (ROC) curve was drawn. The area under the curve (AUC) represents the model's ability to distinguish between outcomes: the closer the AUC is to 1, the more accurately the model correctly classifies the outcomes based on its sensitivity and specificity.

Four hundred and thirty-nine patients (96.9%) had their pre-operative haemoglobin levels checked. One hundred and ninety-five patients (43.0%) had post-operative haemoglobin levels taken. Univariate analysis of pre- to post-operative haemoglobin change therefore excluded those patients who were missing these blood tests. The 14 patients with missing pre-operative haemoglobin levels were excluded from the regression model. Body mass index was not calculable in 139 patients (30.7%), who were missing height or weight from their electronic health records. Therefore, these patients were excluded from this univariate comparison between those who received a blood transfusion and those who did not. All other data recorded in this study were complete.

### Statement of ethics

This study and its database were registered as a clinical audit and approved locally by the West Middlesex University Hospital audit department (audit registration number PCD935). All data were anonymised and collated as part of a clinical audit and therefore patient consent was not required.

## Results

Our data have been made publicly available on Open Science Framework at https://osf.io/5uva4/ ^25^.

Five of the 453 patients (1.1%) received a blood transfusion within 30 days of their operation. Table 1 summarises demographic characteristics of the patients, stratified by whether they received a blood transfusion. On univariate analysis, those who received a transfusion tended to be more co-morbid, as measured by ASA grade ( *p* = 0.017). One patient in each group had an underlying primary haematological malignancy predisposing to bleeding, which was also found to be a risk factor associated with requiring a blood transfusion ( *p* = 0.022): the patient who received a transfusion had myelodysplasia characterised by chronic anaemia and thrombocytopaenia. Similar rates of cardiovascular co-morbidities ( *p* = 1.000) and type 2 diabetes mellitus ( *p* = 0.458) were seen between the two groups.

**Table 1.** Patient demographics stratified by whether they received a blood transfusion. Data are presented as number of patients, followed by the percentage of patients within that transfusion status group in brackets, unless specified otherwise. ASA, American Society of Anesthesiologists; G&S, Group and Save; Hb, Haemoglobin; IQR, InterQuartile Range; NA, Not Applicable; *Mann-Whitney *U* test. †Fisher’s exact test.

Demographic	Blood transfusion received	Blood transfusion not received	*p*-value
**Patients, *n* (% of total)**	5 (1.1)	448 (98.9)	NA
**Median age, years (IQR)**	59 (34 – 67)	50 (39 – 61)	0.703 *
**Gender** ** Female** ** Male**	4 (80.0) 1 (20.0)	308 (68.8) 140 (31.3)	1.000 ^†^
**Median body mass index, kg/m ^2^ (IQR)**	25 (24 – 31)	28 (25 – 32)	0.425 *
**ASA grade** ** I** ** II** ** III** ** IV** ** V**	1 (20.0) 3 (60.0) 0 (0.0) 1 (20.0) 0 (0.0)	128 (28.6) 272 (60.7) 48 (10.7) 0 (0.0) 0 (0.0)	0.017 ^†^
**Co-morbidities** ** Cardiovascular** ** Type 2 diabetes mellitus** ** Primary haematological malignancy** ** Regularly on oral anticoagulant or antiplatelet**	1 (20.0) 1 (20.0) 1 (20.0) 0 (0.0)	134 (29.9) 51 (11.4) 1 (0.2) 33 (7.4)	1.000 ^†^ 0.458 ^†^ 0.022 ^†^ 1.000 ^†^
**Indication** ** Biliary colic** ** Acute cholecystitis** ** Pancreatitis** ** Obstructive jaundice** ** Gallbladder polyps** ** Empyema**	1 (20.0) 3 (60.0) 0 (0.0) 1 (20.0) 0 (0.0) 0 (0.0)	217 (48.4) 132 (29.5) 45 (10.0) 27 (6.0) 12 (2.7) 15 (3.3)	0.301 ^†^
**Operative urgency** ** Emergency** ** Elective**	1 (20.0) 4 (80.0)	80 (17.9) 368 (82.1)	1.000 ^†^
**Operation type** ** Laparoscopic total cholecystectomy** ** Laparoscopic subtotal cholecystectomy** ** Laparoscopic converted to open total cholecystectomy** ** Primary open total cholecystectomy**	4 (80.0) 1 (20.0) 0 (0.0) 0 (0.0)	440 (98.1) 4 (0.9) 3 (0.7) 1 (0.2)	1.000 ^†^
**Median estimated intra-operative blood loss, ml (IQR)**	0 (0 – 50)	0 (0 – 0)	0.056 *
**Median pre-operative Hb, g/l (IQR)**	132 (121 – 145)	134 (126 – 143)	0.651 *
**Median change between pre- and post-operative Hb, g/l (IQR)**	-36 (-68 – -35)	-8 (-14 – -2)	0.001 *
**Number of pre-operative G&S samples** ** 0** ** 1** ** 2**	0 (0.0) 1 (20.0) 4 (80.0)	116 (25.6) 178 (39.5) 154 (34.9)	0.152 ^†^
**Median length of hospital stay, days (IQR)**	8 (5 – 12)	1 (0 – 2)	0.002 *
**Median number of prior emergency hospital attendances** ** with complications of gallstones, *n* (IQR)**	4 (2 – 4)	1 (0 – 1)	< 0.001 *

There was no difference in intra-operative blood loss estimated by the operating surgeon between those who required a blood transfusion and those who did not. However, those patients requiring a blood transfusion had greater drops in post-operative haemoglobin (-36 g/l vs. -8 g/l, *p* = 0.001), despite starting with similar pre-operative haemoglobin levels (132 g/l vs. 134 g/l, *p* = 0.651). Blood transfusions were associated with longer inpatient stays (median 8 days vs. 1 day, *p* = 0.002).

Five procedures (1.1%) were subtotal cholecystectomies. All were undertaken laparoscopically; three on an emergency basis, and two were elective procedures. The indication for surgery for each of these patients was cholecystitis. One of the five patients who underwent subtotal cholecystectomy required a blood transfusion the day after the operation, though patients undergoing a subtotal, rather than total, cholecystectomy were not more likely to require a blood transfusion (p = 0.054).

The patient characteristics and indications for transfusion for each of the five cases who received a blood transfusion are detailed in the Supplementary Table, which can be found at https://osf.io/5uva4/ ^25^. None required emergency group O uncrossmatched blood intra- or post-operatively. At the time of identification of the need for a blood transfusion, each patient was haemodynamically stable; as such, sufficient time would have been present for two G&S samples to be taken and for blood to be fully crossmatched prior to transfusion.

In 116 cases (25.6%), no G&S samples were taken in the preceding five days of the operation. One hundred and seventy-nine patients (39.5%) had one G&S sample taken and 158 (34.9%) had two. Three hundred and one of the 404 patients with ASA grade I or II had at least one G&S sample taken. The cost of analysing a G&S sample in our trust is £11, excluding laboratory staffing costs. Within our cohort, analysis of pre-operative G&S samples therefore cost in the region of £3,800 per year in materials.

Patients who required a peri-operative blood transfusion had more prior emergency hospital attendances in the preceding three years with gallstone complications (median 4 attendances vs. 1 attendance, *p* < 0.001). A logistic regression was performed to determine whether this association held independently of the effects of age, gender, urgency of operation, indication, ASA grade, presence of cardiovascular co-morbidities, type 2 diabetes mellitus, primary haematological malignancy, regular oral anticoagulant or antiplatelet use, or pre-operative haemoglobin level. The regression model was statistically significant ( *p* = 0.042), explained 53.3% (Nagelkerke *R* ^2^) of the variance in blood transfusion requirement and correctly classified 98.9% of cases. Following adjustment for the other co-variants, we found that with each additional emergency hospital attendance, the odds of requiring a blood transfusion increased by a factor of 4.6 (95% confidence interval 1.3 to 16.3, *p* = 0.019). No other co-variants added significantly to the model.

**Figure 1.**
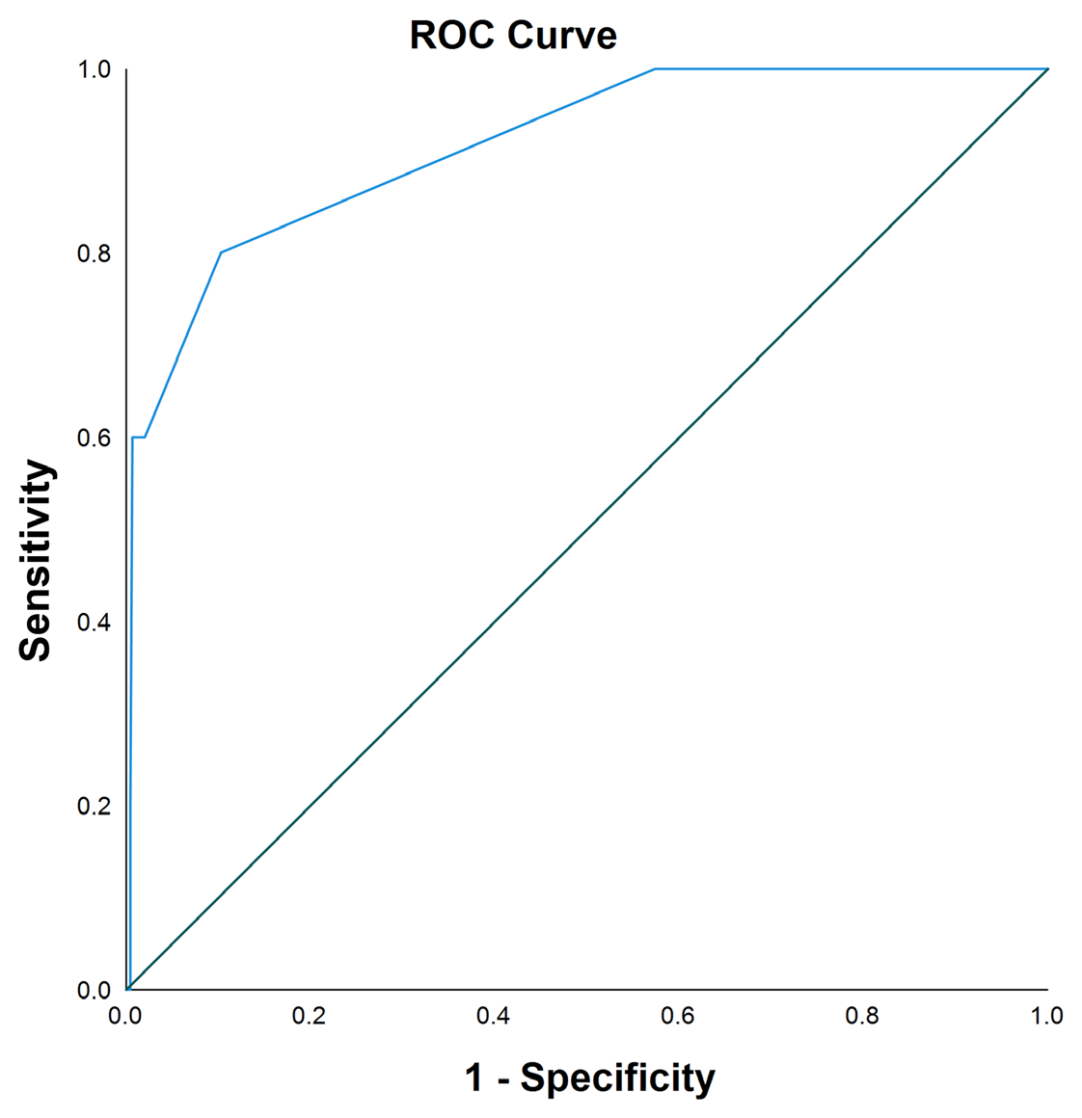
Receiver Operating Characteristic (ROC) curve analysis of number of prior emergency hospital attendances and its impact on the requirement for post-operative blood transfusion. Area under the curve 0.92 (95% confidence interval 0.79 – 1.00).

ROC curve analysis showed an AUC of 0.92 (95% confidence interval 0.79 – 1.00) for number of prior emergency hospital attendances as a predictor of the need for post-operative blood transfusion ( Figure 1). Three or more prior attendances predicted those patients who would require a post-operative blood transfusion with 60.0% sensitivity and 98.0% specificity.

## Discussion

This study demonstrated that, at 1.1%, peri-operative blood transfusion following cholecystectomy in our cohort is rare, and comparable to that in the available literature ^1– 14^. In all our cases, following identification of the need for peri-operative blood transfusion, there was enough time for two G&S samples to be taken and typed, and fully crossmatched blood procured, which corroborates the findings of other cohort studies ^5, 6, 12^. In the rare cases of intra-operative major vascular injury, it would be detrimental to the patient to await crossmatched blood, and therefore even with two valid G&S samples available, rapid transfusion of group O uncrossmatched blood would be required ^1, 6, 12, 14^. G&S testing pre-operatively is therefore not required in most cases ^1– 14^.

We found a strongly independent association between the number of pre-operative emergency attendances with acute gallstone complications and the need for a post-operative blood transfusion following cholecystectomy. Within our cohort, three or more emergency hospital attendances with biliary pathology was highly predictive of this outcome. It is likely that frequent flare-ups of biliary inflammation create more local fibrosis and adhesions, setting up a technically more challenging procedure, as was found in Lo and colleagues’ randomised trial of early versus delayed cholecystectomies ^26^. Fibrosis and oedema in chronic cholecystitis destroy clear operative planes between the gallbladder and the liver bed, which may increase the risk of bleeding from the raw liver surface ^27^. Increased vascular fragility and bleeding may result from the vascular congestion and angiogenesis associated with chronic inflammatory processes ^27, 28^. Four of the 5 patients who required a blood transfusion in our cohort developed post-operative subhepatic haematomas. It may be that these cases were technically challenging, and therefore achieving adequate haemostasis was more difficult; or, even if haemostasis were achieved initially, a high risk of rebleeding followed abdominal closure.

In 1995, Nassar and colleagues published a grading system classifying laparoscopic cholecystectomies according to intra-operative technical difficulty, taking into consideration the density of adhesions and features of the gallbladder and cystic pedicle that make for a more complex dissection ^29^. Their team have shown that patients who undergo emergency laparoscopic cholecystectomy are more likely to have a higher grade of difficulty if they have had multiple hospital admissions with acute biliary disease compared to those for operated on in their first admission, and that a higher Nassar scale grade is associated with a higher risk of bleeding ^30, 31^. Within our cohort, the indication for surgery for all five patients who underwent subtotal cholecystectomies was cholecystitis; three of whom were undergoing emergency procedures for the same. These findings reflect the operative challenges faced when attempting to safely dissect the hepatocystic triangle in a recurrently inflamed gallbladder. This study did not find an association between transfusion of blood products and a subtotal procedure; perhaps decisions to undertake a more conservative procedure were being made before significant bleeding complications occurred.

We found no contingency between estimated intra-operative blood loss and the need for a post-operative blood transfusion. Visual estimation of blood loss is most frequently used as it is quick and effortless, yet notoriously imprecise and poorly reproducible ^32^. Blood collected by suction may be diluted by intra-peritoneal lavage and bile and estimates often do not consider blood contaminating gauze swabs. Accuracy may be improved by strictly measuring the amount of intra-abdominal wash used, and weighing contaminated materials, subtracting their dry weights. That blood loss estimated by the operating surgeon showed no correlation with need for transfusion reinforces the need for clinicians to be Cognisant that blood loss is frequently underestimated.

In one case (Case 3, Supplementary Table, https://osf.io/5uva4/ ^25^), the need for post-operative blood transfusion was attributed to underlying primary haematological malignancy, though our regression model did not show that this was an independent predictor of transfusion. Others have also found primary haematological cancers to be a risk factor for peri-operative transfusion: in an observational study of 4,462 patients in Scotland, this co-morbidity was present in 6 of the 48 (12.5%) cases who required a blood transfusion ^11^. A single-centre retrospective study of 1,167 patients undergoing cholecystectomy in the USA also found that one of their five patients who required a peri-operative blood transfusion had a diagnosis of chronic leukaemia with thrombocytopaenia ^6^. We also found an association between higher ASA grade and need for post-operative transfusion in cholecystectomy, confirming findings by other groups, though this association also did not hold through our regression model ^9, 11^.

There are important limitations that must be considered when interpreting the study findings. Fortunately, blood transfusion is rarely required; as such, with only five patients in our cohort requiring this intervention, it is difficult to definitively exclude those pre-operative risk factors that failed to reach statistical significance due to underpowering. Nevertheless, we believe the relationship between number of acute episodes secondary to gallstone disease and post-operative complications is an important finding favouring performing cholecystectomies on an urgent basis ^2, 8, 9, 13^. A meta-analysis of the published literature may help to confirm the associations between need for blood transfusion and other risk factors identified in other cohort studies, including pre-operative anaemia, obstructive jaundice as an indication for surgery, regular oral anticoagulant use, and underlying type 2 diabetes mellitus ^5, 6, 9, 11, 14^.

The introduction of national UK guidelines that provide recommendations on which patient and operative factors, based on the findings of this and the other studies discussed, would improve selectivity of G&S testing. This would help to prevent uncertainty among staff running pre-operative assessment clinics with regards to appropriate testing, standardise practice between units and avoid wasting of resources.

## Conclusions

Our study suggests that routine G&S testing prior to cholecystectomy, despite this being common practice in many units, is not required. A more selective approach to pre-operative G&S testing can be safe and help save resources. We suggest criteria may include multiple prior emergency hospital attendances, high ASA grade and a history of haematological disorders. It can also be inferred that performing planned cholecystectomy on an urgent basis following emergency admission with acute biliary pathology should be considered where possible to avoid recurrent episodes, as the frequency of prior emergency hospital attendances with gallstone complications may potentially increase the risk of receiving post-operative blood transfusion.

## Data Availability

Open Science Framework: Underlying data for ‘Assessment of routine pre-operative group and save testing in patients undergoing cholecystectomy: a retrospective cohort study’, https://doi.org/10.17605/OSF.IO/5UVA4 ^25^ Data are available under the terms of the Creative Commons Zero “No rights reserved” data waiver (CC0 1.0 Public domain dedication).
