# Supplementary material for: Assessment of routine pre-operative group and save testing in patients undergoing cholecystectomy: a retrospective cohort study
Source: NIHR Open Res. 2024 Oct 23;4:17. Originally published 2024 Apr 5. [Version 2] doi: 10.3310/nihropenres.13543.2 (PMC11519620; doi:10.3310/nihropenres.13543.2)
Supplement: Peer review history

# Reviewer report: Reviewer response for version 2

**DOI:** 10.3310/nihropenres.14935.r33247

**Published:** 2024-10-28

**Authors:** Raimundas Lunevicius

**Affiliations:** 1 Liverpool University Hospitals NHS Foundation Trust,, Lower Lane, Liverpool L9, UK

Assessment of routine pre-operative group and save testing in patients undergoing cholecystectomy: a retrospective cohort study. Version 2.

 Dear Authors,

 The revision of the manuscript has addressed the concerns raised, and questions asked. It significantly enhanced the clarity and quality of the article’s content.

 I am pleased that your manuscript has been accepted for indexing without any reservations.

 Your data are publicly available on Open Science Framework at https://osf.io/5uva4/, which is a real strength of the paper. It makes the study transparent and replicable according to FAIR Principles—GO FAIR.

Is the work clearly and accurately presented and does it cite the current literature?

Yes

If applicable, is the statistical analysis and its interpretation appropriate?

Partly

Are all the source data underlying the results available to ensure full reproducibility?

Yes

Is the study design appropriate and is the work technically sound?

Yes

Are the conclusions drawn adequately supported by the results?

Yes

Are sufficient details of methods and analysis provided to allow replication by others?

Partly

Reviewer Expertise:

Gallbaldder surgery, subtotal cholecystectomy, emergency laparotomy, hernia, global health

I confirm that I have read this submission and believe that I have an appropriate level of expertise to confirm that it is of an acceptable scientific standard.

# Reviewer report: Reviewer response for version 2

**DOI:** 10.3310/nihropenres.14935.r33248

**Published:** 2024-10-25

**Authors:** Yulia Lin

**Affiliations:** 1 University of Toronto, Toronto, ON, Canada

The authors have addressed my comments. No further comments.

Is the work clearly and accurately presented and does it cite the current literature?

Yes

If applicable, is the statistical analysis and its interpretation appropriate?

Partly

Are all the source data underlying the results available to ensure full reproducibility?

Yes

Is the study design appropriate and is the work technically sound?

Yes

Are the conclusions drawn adequately supported by the results?

Partly

Are sufficient details of methods and analysis provided to allow replication by others?

Yes

Reviewer Expertise:

Transfusion Medicine clinical practice, education and quality improvement

I confirm that I have read this submission and believe that I have an appropriate level of expertise to confirm that it is of an acceptable scientific standard.

# Reviewer report: Reviewer response for version 1

**DOI:** 10.3310/nihropenres.14701.r31980

**Published:** 2024-09-15

**Authors:** Raimundas Lunevicius

**Affiliations:** 1 Liverpool University Hospitals NHS Foundation Trust,, Lower Lane, Liverpool L9, UK

The authors defined the local audit project as a retrospective cohort study that explored the frequency of testing in the general surgery unit, rates of and independent pre-operative risk factors for peri-operative blood transfusion. The authors have decided to conduct this study because the United Kingdom (UK) lacks national guidance recommending which patient or procedural factors warrant pre-operative G&S testing, although it has been shown, between 1999 and 2022, that G&S testing is overperformed before laparoscopic cholecystectomy in various health care facilities of the UK, and, therefore, a targeted approach should be adopted. The authors also emphasised that the decision to perform G&S testing relies on the clinical judgement of staff running pre-operative assessment clinics, i.e. on local resources and standard operative procedures.

 Purported pre-operative risk factors for peri-operative blood transfusion in the available literature include anaemia, regular oral anticoagulant use, cardiovascular co-morbidities, type 2 diabetes mellitus, primary haematological malignancies and increased American Society of Anesthesiologists (ASA) Grade. Whether these associations are independent predictors (incl. primary haematological malignancies) of blood transfusion is unclear.

 Therefore, the study aimed to identify independent pre-operative risk factors that may predict peri-operative blood transfusion in these patients. The authors correctly hypothesised (every gallbladder surgeon knows that)  that the odds of transfusion could be predicted by the number of prior emergency attendances to the hospital with acute biliary pathology secondary to gallstones. In this regard, they are proud to be the first (the phrase “we are the first” used) to explore this association, adding a unique perspective to the existing literature.

 Methods

- Study methodology. I find that ’this cross-sectional study analysed 453 consecutive adult patients’. On the other hand, this study suggests that patients’ electronic hospital records were analysed to extract and abstract the information into data points. Please consider rephrasing.
- I find ‘Patients aged under 16 years were not included in the study’. Please specify (section ‘Results’) how many patients you have excluded’. If none, please delete this phrase. In addition, ‘patients aged under 16 years’. Is it a correct writing?
- Patients were identified by their International Classification of Diseases, 10th revision codes. This is unspecific writing. It remains unclear how this was done: by the authors or IT professionals. Also, ICD-10 codes could have been provided clearly (please do it). I am writing this because methods and data should be well-described so that they can be replicated and/or combined in different settings. That is a fundamental FAIR principle, https://www.go-fair.org/fair-principles/.
- The paragraph “Length of hospital stay referred to the number of post-operative days during the admission in which the operation took place. It did not include any prior admissions nor post-operative readmissions.” The second sentence is highly unusual. I would recommend deleting it.   
- Re “Estimated intra-operative blood loss was recorded by the operating surgeon in the operation note.” It is the most inaccurate and most diseased method of estimation of blood loss. It needs to be discussed in ‘Discussion’.
- Re ‘post-operative haemoglobin was the lowest value within 30 days of the operation’. I don’t understand it (the lowest value). Please clarify.
- I find the phrase ‘All other data recorded in this study were complete.’ However, the Open Science Framework at https://osf.io/5uva4/ shows that this is not the case. Many empty cells within the Excel database (for example, cell H2, cell H29, Cell h44, etc.) indicate that not all data recordings in this study were completed. Please consider this point seriously and update the Open Science Framework accordingly (it is a pretty problematic task; you will need to communicate with the administrator of the Open Science Framework as a new dataset will generate a new DOI).
- Statistical analysis. It would be useful to understand why the authors have shown the number of degrees of freedom in brackets. Please add the sentence and explain to the readers (including the patients and public) what that would mean – one degree of freedom or another. I say ‘the patients and public’ because the first subsection of Methods concerns the patients and public. For example, what does it mean (χ2 (3) = 90.3, p < 0.001)? I mean χ2 (3) = 90.3.
- I find that ‘Statistical analyses of categorical data were performed using Pearson’s chi-squared test. Also, I find the comparisons between 2 subgroups - n= 5 and n= 448. Please explain in the methods why you had chosen the chi-squared test instead of the exact Fisher’s test when one of the subgroups included only 5 patients. Would you consider conducting a new analysis with a more precise test in use?
- The area under the curve (AUC) can be used to visualise the sensitivity and specificity of a model predicting a certain outcome: the closer the AUC is to 1. That is correct; however, why do the authors use the verb form ‘can be’? The authors ‘used to visualise’ (not ‘can be used to visualise’). Please revise the paper from an academic language point of view.

Results

- I find 28 patients with obstructive jaundice (1 + 27, Table 1). This has not been said in Methods. Please clarify.
- It is unusual to see no subtotal cholecystectomies in > 453 patients with such indications for surgery. Please provide a comment (Discussion). The source: Lunevicius R, et al., 2022 [Ref-1]
- Figure 1. Please specify Y-axis (Sensitivity)
- The phrase ‘statistically significant is old-fashioned. Please speak about associations in your paper (you did it in Discussion – excellent writing)
- I find the ‘Supplementary Table’. This needs to be linked to the Supplementary Table source online.

Discussion

- Please avoid writing this kind of ‘we are the first to demonstrate’. It doesn’t look good (especially in the context of your paper). Others will decide who is the first or the second (a higher Nassar scale grade is associated with a higher risk of bleeding)
- Future prospective national studies with a larger sample size are required to further assess whether G&S routine testing is required and its associated safety. So, I am inclined to say that prospective national studies are unnecessary in light of references 1 – 14 and the results and findings of this study.
- The point (rationale of the study) mentioned in the Introduction (“The authors have decided to conduct this study because the United Kingdom (UK) lacks national guidance recommending which patient or procedural factors warrant pre-operative G&S testing, although it has been shown, between 1999 and 2022, that G&S testing is overperformed before laparoscopic cholecystectomy in various health care facilities of the UK, and, therefore, a targeted approach should be adopted. The authors also emphasised that the decision to perform G&S testing relies on the clinical judgement of staff running pre-operative assessment clinics, i.e. on local resources and standard operative procedures) and requires discussion in light of the findings of this study.

Is the work clearly and accurately presented and does it cite the current literature?

Yes

If applicable, is the statistical analysis and its interpretation appropriate?

Partly

Are all the source data underlying the results available to ensure full reproducibility?

Yes

Is the study design appropriate and is the work technically sound?

Yes

Are the conclusions drawn adequately supported by the results?

Yes

Are sufficient details of methods and analysis provided to allow replication by others?

Partly

Reviewer Expertise:

Gallbaldder surgery, subtotal cholecystectomy, emergency laparotomy, hernia, global health

I confirm that I have read this submission and believe that I have an appropriate level of expertise to confirm that it is of an acceptable scientific standard, however I have significant reservations, as outlined above.

## References

[1] : A nationwide analysis of gallbladder surgery in England between 2000 and 2019. *Surgery* .2022;171(2) : 10.1016/j.surg.2021.10.025 276-284 10.1016/j.surg.2021.10.025 34782153

# Response

**Published:** 2024-09-23

**Authors:** Lawrence O'Leary

**Affiliations:** University of Liverpool, Liverpool, England, UK

*Dear Mr Lunevicius,*

*Thank you for taking the time to read our manuscript. We really appreciate your advice and helpful suggestions to improve the paper. We have responded to each of your comments in turn below, and hope you approve of our changes.*

*Yours sincerely,*

*Mr Lawrence O’Leary, on behalf of the authors*

 Methods

- Study methodology. I find that ’this cross-sectional study analysed 453 consecutive adult patients’. On the other hand, this study suggests that patients’ electronic hospital records were analysed to extract and abstract the information into data points. Please consider rephrasing.

*Thank you. We have rephrased the beginning of the section so it more clearly specifies that it was a retrospective cohort study analysing patients’ electronic health records.*

- I find ‘Patients aged under 16 years were not included in the study’. Please specify (section ‘Results’) how many patients you have excluded’. If none, please delete this phrase. In addition, ‘patients aged under 16 years’. Is it a correct writing?

*As you suggest, we have deleted this sentence. We have said the analysis was of adult patients in the first paragraph. No patients were excluded from the analysis.*

- Patients were identified by their International Classification of Diseases, 10th revision codes. This is unspecific writing. It remains unclear how this was done: by the authors or IT professionals. Also, ICD-10 codes could have been provided clearly (please do it). I am writing this because methods and data should be well-described so that they can be replicated and/or combined in different settings. That is a fundamental FAIR principle, https://www.go-fair.org/fair-principles/.

*Thank you. The database was created by IT professionals from the local audit department and sent to the authors. What was assumed to be ICD-10 codes were, in fact, OPCS-4 procedure codes. We have now specified this in the second paragraph of the section with the codes they used to compile the list of patients. We apologise for this error.*

- The paragraph “Length of hospital stay referred to the number of post-operative days during the admission in which the operation took place. It did not include any prior admissions nor post-operative readmissions.” The second sentence is highly unusual. I would recommend deleting it.   

*We have deleted the paragraph.*

- Re “Estimated intra-operative blood loss was recorded by the operating surgeon in the operation note.” It is the most inaccurate and most diseased method of estimation of blood loss. It needs to be discussed in ‘Discussion’.

*Thank you. We included this, as visual estimation is the most commonly used method of estimating and recording intraoperative blood loss. As you kindly suggest, we have added a paragraph highlighting its inaccuracy, and that our study reinforces that surgeons’ perceived blood loss does not correlate with risk of needing transfusion.*

- Re ‘post-operative haemoglobin was the lowest value within 30 days of the operation’. I don’t understand it (the lowest value). Please clarify.

*Several patients had post-operative blood tests taken on multiple occasions. If this was the case, for the sake of our analysis, we defined “post-operative haemoglobin” as the lowest haemoglobin level. We have clarified this in the manuscript.*

- I find the phrase ‘All other data recorded in this study were complete.’ However, the Open Science Framework at https://osf.io/5uva4/ shows that this is not the case. Many empty cells within the Excel database (for example, cell H2, cell H29, Cell h44, etc.) indicate that not all data recordings in this study were completed. Please consider this point seriously and update the Open Science Framework accordingly (it is a pretty problematic task; you will need to communicate with the administrator of the Open Science Framework as a new dataset will generate a new DOI).

*Thank you. We sincerely apologise for this inaccuracy. We have updated the manuscript under “Statistical analysis” to highlight the missing values in pre- and post-operative haemoglobin and body mass index, and explained how we have managed these in our analyses.*

- Statistical analysis. It would be useful to understand why the authors have shown the number of degrees of freedom in brackets. Please add the sentence and explain to the readers (including the patients and public) what that would mean – one degree of freedom or another. I say ‘the patients and public’ because the first subsection of Methods concerns the patients and public. For example, what does it mean (χ2 (3) = 90.3, p < 0.001)? I mean χ2 (3) = 90.3.

*Thank you. After some deliberation, we have decided that showing the chi-squared values and degrees of freedom are complicating the readability of the manuscript without adding value to the messages we are trying to get across. We have therefore opted to exclude these and keep the *p *-values only.*

- I find that ‘Statistical analyses of categorical data were performed using Pearson’s chi-squared test. Also, I find the comparisons between 2 subgroups - n= 5 and n= 448. Please explain in the methods why you had chosen the chi-squared test instead of the exact Fisher’s test when one of the subgroups included only 5 patients. Would you consider conducting a new analysis with a more precise test in use?

*Thank you. You are quite right that the Fisher’s exact test would have been more accurate for the analysis of categorical data. We have re-run the analyses for all those comparisons in which we had previously used Pearson’s chi-squared tests. We have updated the methodology and *p *-values in Table 1, in the body of the Results section and the Abstract accordingly. Those results that were previously statistically significant with Pearson’s chi-squared held as being statistically significant using Fisher’s; and those that did not reach statistically significance remained insignificant.*

- The area under the curve (AUC) can be used to visualise the sensitivity and specificity of a model predicting a certain outcome: the closer the AUC is to 1. That is correct; however, why do the authors use the verb form ‘can be’? The authors ‘used to visualise’ (not ‘can be used to visualise’). Please revise the paper from an academic language point of view.

*Thank you. This sentence has been changed to “The area under the curve (AUC) represents the model's ability to distinguish between outcomes: the closer the AUC is to 1, the more accurately the model classifies the outcomes based on its sensitivity and specificity.”*

 Results

- I find 28 patients with obstructive jaundice (1 + 27, Table 1). This has not been said in Methods. Please clarify.

*These 28 patients comprised 19 patients who underwent elective cholecystectomy and 9 emergency “hot” operations. They all presented with obstructive jaundice and were investigated with MRCP and, if choledocholithiasis was confirmed, underwent removal of common bile duct stones with ERCP. Only after the CBD had been cleared did the patients undergo cholecystectomy. To clarify this, in the Methods section, we have added the following sentence: “Any patient who presented with obstructive jaundice was investigated with magnetic resonance cholangiopancreatography and, if required, underwent clearance of obstructing choledocholithiasis with endoscopic retrograde cholangiopancreatography prior to cholecystectomy.”*

- It is unusual to see no subtotal cholecystectomies in > 453 patients with such indications for surgery. Please provide a comment (Discussion). The source: Lunevicius R, et al., 2022 [Ref-1]

*In our results, we did not divide the procedures into total and subtotal procedures; rather, we described the operative approach as laparoscopic, open or converted, anticipating that the size of incision and need to convert might contribute to the risk of blood loss and therefore need for transfusion. For clarity, we have relooked at the operation undertaken, and identified that 5 patients (1%) underwent subtotal procedures, all completed laparoscopically, and have added these to the “Operation type” heading in Table 1. We have added this as a new heading in the available data and reuploaded to the Open Science Framework, as well as adding a paragraph to the Results table. Patients who had a subtotal, rather than total, cholecystectomy were not more likely to receive a blood transfusion in our cohort. We have discussed these findings in the paragraph related to Nassar’s grading system and the association between operative difficulty increasing risk of bleeding.*

- Figure 1. Please specify Y-axis (Sensitivity)

*Thank you. The y-axis label was inadvertently missed out. This has been reuploaded with the axis title.*

- The phrase ‘statistically significant is old-fashioned. Please speak about associations in your paper (you did it in Discussion – excellent writing)

*Thank you for this advice. We have changed the phrasing when this occurred.*

- I find the ‘Supplementary Table’. This needs to be linked to the Supplementary Table source online.

*Thank you. We have added the URL to our Open Science Framework project following mentions of the Supplementary Table. Here can be found both the Supplementary Table and our anonymised data.*

 Discussion

- Please avoid writing this kind of ‘we are the first to demonstrate’. It doesn’t look good (especially in the context of your paper). Others will decide who is the first or the second (a higher Nassar scale grade is associated with a higher risk of bleeding)

*Thank you, we have removed this on your advice.*

- Future prospective national studies with a larger sample size are required to further assess whether G&S routine testing is required and its associated safety. So, I am inclined to say that prospective national studies are unnecessary in light of references 1 – 14 and the results and findings of this study.

*We have rephrased our concluding paragraph in the Discussion.*

- The point (rationale of the study) mentioned in the Introduction (“The authors have decided to conduct this study because the United Kingdom (UK) lacks national guidance recommending which patient or procedural factors warrant pre-operative G&S testing, although it has been shown, between 1999 and 2022, that G&S testing is overperformed before laparoscopic cholecystectomy in various health care facilities of the UK, and, therefore, a targeted approach should be adopted. The authors also emphasised that the decision to perform G&S testing relies on the clinical judgement of staff running pre-operative assessment clinics, i.e. on local resources and standard operative procedures) and requires discussion in light of the findings of this study.

*Thank you, we have now brought our Discussion back round to the need for explicit national guidelines to help standardise G&S testing based on our findings and those of other studies.*

# Reviewer report: Reviewer response for version 1

**DOI:** 10.3310/nihropenres.14701.r31977

**Published:** 2024-06-10

**Authors:** Yulia Lin

**Affiliations:** 1 University of Toronto, Toronto, ON, Canada

Thank you for the opportunity to review this well-written manuscript. This is a retrospective audit of 453 patients undergoing cholecystectomy at two teaching hospitals over 18 months. The authors found that despite 74% of patients having a preoperative group and save (G&S) performed in the 5 days prior to surgery, only 5 pts (1.1%) required a blood transfusion. These transfusions were non-urgent and occurred postoperatively where there would have been adequate time to perform a G&S. Logistic regression demonstrated an association between prior emergency attendance. Univariable analyses showed an association of underlying haematological malignancy and higher ASA with blood transfusion that was not present in logistic regression. The cost of the unnecessary G&S tests was £3800 per year.

 There are clarifications that would be helpful to the reader:

 Methods

1. Please clarify that this was a “retrospective” cross-sectional study.
2. Please clarify whether transfusion data were obtained from the electronic record linked to the transfusion laboratory information system as chart records that are not linked can be inaccurate.
3. This is a comment for the authors’ institution. Many institutions allow a group and save to be done within 30 days of the procedure (rather than only 5 days) provided that the patient has not received a transfusion or been pregnant in the 3 months prior. This may increase efficiency for patients who do require a G&S.

Results

1. Please clarify the hospital policy for type of blood for emergency uncrossmatched blood. Most institutions would provide group O positive blood and only group O negative to individuals of childbearing potential. Depending on the hospital policy, the text should be revised to group O uncrossmatched blood. This change is suggested to avoid the common misconception that only group O negative can be given in an emergency uncrossmatched scenario.  (This also applies to the reference to group O negative in the Discussion).
2. Please clarify if the cost of the preoperative G&S tests includes only the ASA grade I and II patients or all G&S done preoperatively for this cohort.
3. In the logistic regression, the only independent factor that was associated with perioperative blood transfusion was prior emergency hospital attendance. It should be stated that the association of higher ASA and hematological malignancy was only seen in univariable analysis in the Abstract.

Discussion

1. The primary question of the study was the frequency of G&S testing and perioperative blood transfusion as noted in the first sentence of the conclusion. Suggest that paragraph 6 of the Discussion be the first paragraph summarizing the main findings of the study.
2. While the association of previous emergency attendance was noted in this study, the sample size of events (patients who received a blood transfusion) is only 5. Any statistical associations found (or not found) are limited given that there are so few events. Please add this major limitation.

Conclusion

1. Because patients who has prior emergency attendance had greater risk of blood transfusion, does not necessarily translate to those patients requiring surgery on their emergency attendance. Suggest de-emphasizing this inference. (This also applies to the final paragraph in the Plain English summary)
2. It would be reasonable to suggest potential criteria for G&S e.g. prior emergency hospital attendance, higher ASA and known history of haematological malignancy.

Is the work clearly and accurately presented and does it cite the current literature?

Yes

If applicable, is the statistical analysis and its interpretation appropriate?

Partly

Are all the source data underlying the results available to ensure full reproducibility?

Yes

Is the study design appropriate and is the work technically sound?

Yes

Are the conclusions drawn adequately supported by the results?

Partly

Are sufficient details of methods and analysis provided to allow replication by others?

Yes

Reviewer Expertise:

Transfusion Medicine clinical practice, education and quality improvement

I confirm that I have read this submission and believe that I have an appropriate level of expertise to confirm that it is of an acceptable scientific standard, however I have significant reservations, as outlined above.

# Response

**Published:** 2024-09-23

**Authors:** Lawrence O'Leary

**Affiliations:** University of Liverpool, Liverpool, England, UK

Dear Dr Lin,

 We really appreciate you taking the time to review our article and thank you for your expertise and advice on how we can make our messages clearer. We have responded to each of your suggested clarifications in turn below.

 Yours sincerely,

 Mr Lawrence O’Leary, on behalf of the authors

**1. Reviewer:** Methods - Please clarify that this was a “retrospective” cross-sectional study.

***Author Response: **Thank you – we have added this important detail to both the main manuscript Methods section and the abstract.*

**2. Reviewer:** Please clarify whether transfusion data were obtained from the electronic record linked to the transfusion laboratory information system as chart records that are not linked can be inaccurate.

***Author Response: **In our institution, the patient electronic records are automatically updated with product type and volume by the transfusion laboratory when blood products are released to the wards. We primarily used this information to identify when blood products were prescribed, and confirmed these were administered by cross-referencing patient fluid balance charts. We have clarified this in the Methods section as you kindly suggest.*

**3. Reviewer: **This is a comment for the authors’ institution. Many institutions allow a group and save to be done within 30 days of the procedure (rather than only 5 days) provided that the patient has not received a transfusion or been pregnant in the 3 months prior. This may increase efficiency for patients who do require a G&S.

***Author Response: **Thank you for this comment. We have checked with our transfusion laboratory, and confirmed they allow group and saves within 30 days of the procedure provided the same criteria you list are met. We have fed this back to our department.*

**4. Reviewer: **Results - Please clarify the hospital policy for type of blood for emergency uncross matched blood. Most institutions would provide group O positive blood and only group O negative to individuals of childbearing potential. Depending on the hospital policy, the text should be revised to group O uncross matched blood. This change is suggested to avoid the common misconception that only group O negative can be given in an emergency uncross matched scenario.  (This also applies to the reference to group O negative in the Discussion).

***Author Response: **We have confirmed this is the case in our unit and as per our national British Society for Haematology guidelines and corrected the manuscript accordingly.*

**5. Reviewer: **Please clarify if the cost of the preoperative G&S tests includes only the ASA grade I and II patients or all G&S done preoperatively for this cohort.

***Author Response: **The costing was for the whole cohort, and we have amended this to make this clearer.*

**6. Reviewer: **In the logistic regression, the only independent factor that was associated with perioperative blood transfusion was prior emergency hospital attendance. It should be stated that the association of higher ASA and hematological malignancy was only seen in univariable analysis in the Abstract.

***Author Response: **Thank you – this has been clarified in the Abstract.*

**7. Reviewer: **Discussion - The primary question of the study was the frequency of G&S testing and perioperative blood transfusion as noted in the first sentence of the conclusion. Suggest that paragraph 6 of the Discussion be the first paragraph summarizing the main findings of the study.

***Author Response: **We agree with this suggestion, and have rearranged the Discussion section as per your advice.*

**8. Reviewer: **While the association of previous emergency attendance was noted in this study, the sample size of events (patients who received a blood transfusion) is only 5. Any statistical associations found (or not found) are limited given that there are so few events. Please add this major limitation.

***Author Response: **Thank you – we have made the Limitations paragraph clearer to reflect this.*

**9. Reviewer: **Conclusion - Because patients who has prior emergency attendance had greater risk of blood transfusion, does not necessarily translate to those patients requiring surgery on their emergency attendance. Suggest de-emphasizing this inference. (This also applies to the final paragraph in the Plain English summary)

***Author Response: **Our intention was not to infer that these patients should necessarily have a “hot” emergency cholecystectomy; rather, that these elective operations should be treated as urgent, and delays to operations avoided. We have therefore clarified this in the Plain English summary and Conclusion by adding that delays to *planned *operations should be avoided.*

**10. Reviewer: **It would be reasonable to suggest potential criteria for G&S e.g. prior emergency hospital attendance, higher ASA and known history of haematological malignancy.

***Author Response: **Thank you, we have added this suggestion.*
